# Causes of Death in Implant Patients Treated in the Edentulous Jaw: A Comparison between 2098 Deceased Patients and the Swedish National Cause of Death Register

**DOI:** 10.1155/2019/7315081

**Published:** 2019-03-11

**Authors:** Jan Kowar, Victoria Stenport, Mats Nilsson, Torsten Jemt

**Affiliations:** ^1^Prosthodontist, Brånemark Clinic, Public Dental Health Care Service, Gothenburg, Västra Götaland, Sweden; ^2^Associate Professor, Department of Prosthetic Dentistry/Dental Material Science, The Sahlgrenska Academy at Göteborg University, Gothenburg, Sweden; ^3^Statistician/Epidemiologist, Futurum, Academy of Health and Care, Jönköping and Department of Medical and Health Sciences, Linköping University, Linköping, Jönköping, Sweden; ^4^Professor, Department of Prosthetic Dentistry/Dental Material Science, The Sahlgrenska Academy at Göteborg University, Gothenburg, Sweden

## Abstract

**Background:**

Previous research has reported an association between tooth loss and patient mortality, while the cause of death has not been elucidated.

**Objective:**

The purpose was to describe and compare the cause of death in implant patients treated consecutively in the edentulous arch with a reference population.

**Methods:**

Altogether, 3902 patients were included between 1986 and 2014. Data on the causes of death for deceased patients were compared to the Swedish National Cause of Death Register for a comparable time period. Standardised mortality ratios (SMRs) were calculated based on gender and age and tested for statistical significance.

**Results:**

Most deceased patients (2,098) died from diseases in the circulatory system (CVD; 42%) and from cancers (26%). SMR indicated a generally increased mortality (total group) compared to the reference population during inclusion (*P* < 0.05; 1986–2014). Patients treated early (1986–1996) showed a lower SMR compared to patients treated later (*P* < 0.05; 1997–2014) especially related to CVDs. Younger patients (<60 years at surgery) showed an increased mortality due to CVDs when treated late (1997–2014; SMR = 5.4, *P* < 0.05). Elderly patients (>79 years at surgery) showed a significantly lower mortality in almost all observed causes of death (1986–2014; *P* < 0.05) with also a significantly lower mortality due to CVDs during the early period (1986–1996; SMR = 0.3, *P* < 0.05).

**Conclusion:**

An overall increased mortality was observed for the edentulous implant patient compared to the reference population. Elderly patients (>79 years) showed significantly lower mortality for all causes of death independent of the time period of implant surgery. Younger patients (<60 years) present an increased risk for early mortality related to CVD. SMR for all causes of death increased for patients treated late (1997–2014) as compared to patients treated early (1986–1996).

## 1. Introduction

The prevalence of tooth loss and edentulism has been decreasing in most European countries during the last decades [1]. Oral rehabilitation with implant-supported prostheses is a common treatment and known to improve oral function and quality of life [2]. Furthermore, the time span between when the patient become edentulous and time of implant rehabilitation has shortened remarkably over the last decades [3]. Nowadays, the rehabilitation often starts immediately after tooth loss [3] while in the 1990's the average time of edentulousness was over 14 years before implant treatment [4].

The major reasons for tooth loss are caries and periodontitis [5, 6]. The latter is described as a chronic infection in the supportive tissue around the teeth. There is some hypothesis that chronic inflammation in the periodontium contributes to the pathogenesis of diseases in the circulatory system which could proceed to mortality due to diseases in the circulatory system [7], but a causality has not been shown.

Furthermore, the number of remaining teeth has been shown to be a predictor for all-cause and cardiovascular mortality [8–10]. The degree of tooth loss also seems to be associated with an increased risk for peri-implant complications in both short- and long-term follow-up studies [11, 12]. Thus, risk for early severe peri-implant inflammation after the implant placement has been reported to be associated with the middle-aged patient [13], and early total tooth loss has been suggested to be a risk factor in relation to mortality [14]. Similarly, early placement of artificial prostheses replacing the knee and hip joints have also been reported to be associated with an increased risk for early mortality in young (<55 years) patients [15, 16]. Edentulous patients, treated with dental implants before the age of 60 years, presented a higher mortality rate compared to a reference population, in contrast to elderly edentulous implant patients who live longer than the reference population [14]. The reasons for these observations are still unclear.

The aim of this study was to examine and report the pattern of cause of death in a consecutive group of implant patients treated in the edentulous arch at one referral clinic and to compare the results to the mortality pattern in the entire Swedish population in different age groups.

## 2. Materials and Methods

The present study is a retrospective study including all consecutive patients treated in the edentulous jaw between January 1986 and December 2014 at one clinic (Brånemark clinic, Public Dental Health Service, Gothenburg, Sweden). The patients were provided with dental implants (Brånemark implant, Nobel Biocare AB, Sweden) either in the upper or lower edentulous jaw or in both. The study was approved by the local Ethics Committee in Gothenburg (#460–15).

### 2.1. Study Population

A total of 9037 patients were treated with a total of 40937 implants during 11551 operations at the clinic during the inclusion period. Patients who were not residents in Sweden or who moved abroad during the follow-up period were excluded before final inclusion. Altogether, 3,902 patients (43.2%) remained for inclusion, who all were edentulous in the upper and/or lower jaw and provided with implant-supported prostheses. If the patient was treated in both jaws, only the first event of surgery was included.

The total group of included edentulous patients (*N*=3902 patients) was divided into three subgroups according to patients' age at time of the first implant surgery. The first group includes 1,262 patients (54% females) who were younger than 60 years, the second group comprised 2,350 patients (55% females) between an age of 60 and 79 years, and the third subgroup comprised 290 elderly patients (62% females), aged 80 years or older. Altogether, 2142 and 1760 of the included patients were females and males, respectively.

**Figure 1:**
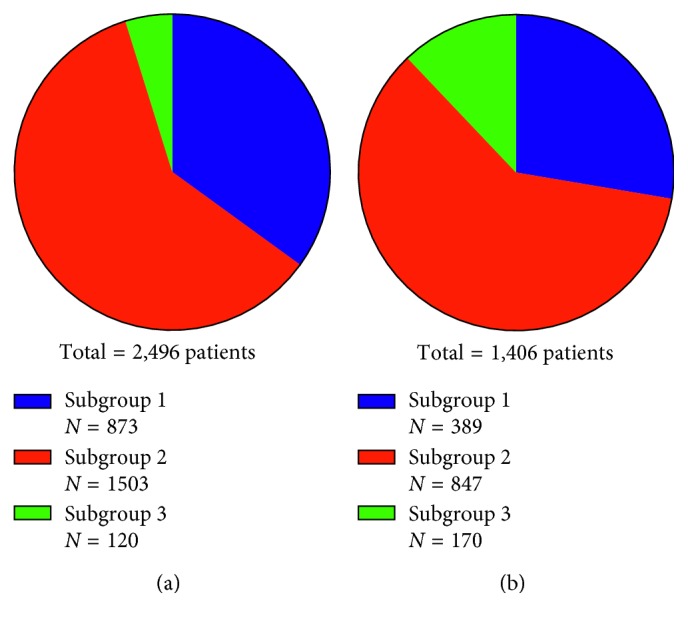
The total group of 3902 included patients was arranged into two subgroups with regard to time at surgery ((a) 1986 to 1996; (b) 1997 to 2014). Distribution of number of treated implant patients according to age is presented for the two subgroups (subgroup #1, <60 years at implant surgery; subgroup #2, 60 to 79 years; subgroup #3, >79 years).

In addition to the age-specific subgroups, the entire study population (*N*=3902 patients) was also divided into two time-specific groups where the first group includes all patients treated between 1986 and 1996 and the second group covers all edentulous patients treated with dental implants between 1997 and 2014. The distribution of the three age-related subgroups in this two time periods of inclusion is shown in Figure 1.

All included patients (*N*=3902 patients) were available for follow-up in the Swedish national population registers from implant treatment to the termination of the study. Data on the cause of death for all deceased patients were obtained from the Swedish National Cause of Death Register of the National Board of Health and Welfare in Sweden. In this register, the date of death and underlying cause of death was registered for all citizens in Sweden and cause of death was coded using the International Classification of Diseases (ICD), version 10 [17]. When ICD-8 or ICD-9 codes were applied, they have been translated into ICD-10 codes using the conversion table from the National Board of Health and Welfare. The observed number of deceased patients in the study group was compared with an expected number based on the official mortality register of the Swedish population. Age, gender, and the calendar year-specific mortality rates from the Swedish National Cause of Death Register was the basis for the calculation of the expected number of deceased patients. Until 1996, the expected number of deceased patients was calculated using the statistical database for causes of death from the Statistics Sweden [18] and from 1997 using a similar database from the National Board of Health and Welfare [19]. Standardised mortality ratios (SMRs) for the populations were calculated by using the ratio between observed deaths in the study group and expected deaths in the reference population.

### 2.2. Statistical Analyses

In the present report, descriptive data are presented as numbers, frequencies, percentages, medians, and 25^th^ (*P*_25_) and 75^th^ (*P*_75_) percentiles. In general, numbers are corrected to one decimal in tables and text. Patients were grouped according to age, different for different types of analysis. Demographic data and number of deaths were obtained from Statistics Sweden and the National Board of Health and Welfare for the Swedish population (used as reference population). This was used to calculate mortality in the age-specific reference groups and to calculate the standardised mortality rate (SMR, observed number of deaths divided by the expected number of deaths, standardised to the Swedish population) for the investigated causes of death. For SMR, 95% confidence intervals were calculated, and the SMR was considered to be statistically significant if the 95% confidence interval did not contain one (1.00). The calculated *P* values were considered statistically significant if *P* < 0.05 with 95% confidence intervals. The statistical calculation was performed using SAS/STAT® software using the procedure PROC STDRATE, ©SAS Institute Inc., Cary, NC, USA.

## 3. Results

Out of a total of 3,902 included edentulous patients, 2,098 (53.8%) patients were deceased during the inclusion period (1986–2014). The median age at death for the 1,000 men (48%) and 1,098 women (52%) was 69 years (*P*_25_=62 years and *P*_75_=75 years).

Among the 2,098 deceased patients, a total of 883 (42%) patients died of diseases in the circulatory system, while 556 (26%) patients died due to cancers. In the total Swedish reference population (>45 years), mortality related to diseases in the circulatory system accounted for almost 50 percentage of cause of deaths, followed by various cancers causing 25% of deaths. For the total study group, the standardised mortality ratio (SMR) indicated a generally increased mortality compared to the reference population (Table 1). However, patients treated between 1986 and 1996 showed a lower SMR compared to those treated in the later period between 1997 and 2014, especially in relation to diseases in the circulatory system (Tables 2 and 3). As shown in Table 3, the group of patients treated in the later time period (1997–2014) has a significantly increased SMR in almost all causes of death independent of gender.

### 3.1. Causes of Death in Patients Younger than 60 Years at Implant Operation

In contrast to the overall adult Swedish population, cancers are the most common cause of death in the present age group ranging from 45 to 59 years. The cause-specific mortality in the subgroup of 1,262 patients who were <60 years of age at the time of implant surgery shows a generally increased SMR (SMR = 1,6; *P* < 0.05). Seventy-five patients (5.9%) were deceased during the inclusion period. The most frequently observed causes of death were diseases of the circulatory system (*N*=21) and cancer (*N*=26), including one-third of all deceased patients each. Significant differences for the deceased patients were observed between the early (1986–1996) and late (1997–2014) time period, where the latter shows increased values for SMR in all causes of death (SMR = 1.6 and 2.7, respectively). It can be noticed that the number of deaths related to diseases of the circulatory system in patients treated between 1997 and 2014 was over five times higher as compared to the general population (*P* < 0.05; Table 4). An increased mortality from diseases in the respiratory system as well as from mental illness and endocrine and metabolic diseases was apparent in this subgroup of patients. However, significant differences could not be obtained.

### 3.2. Causes of Death in Patients 60–79 Years of Age at Implant Operation

In this subgroup that included 2,350 individuals, 1,497 patients (63.7%) died during the time of inclusion. Most of the patients (42%) died because of diseases in the circulatory system followed by tumours (27%) and diseases in the respiratory system (8%). For all causes of death, the observed number of deceased patients is lower than expected (SMR = 0.9, *P* < 0.05). No clear difference was detected between patients treated between 1986 and 1996 and those treated between 1997 and 2014 (*P* > 0.05). However, mortality due to mental illness was two and half times higher in the total study group (1986–2014) as compared to the reference population (*P* < 0.05). Also, a higher risk for mortality due to diseases in the respiratory system was found in this group (SMR = 1.4, *P* < 0.05).

### 3.3. Causes of Death in Patients Older than 79 Years at Implant Operation

Fifty percent of the elderly, aged 80 years or more, are expected to die due to diseases in the circulatory system in the Swedish population. Altogether, 236 patients in the present elderly group (>79 years) died during inclusion (81.4%) between 1986 and 2014. However, the mortality was still significantly lower than expected (SMR = 0.6, *P* < 0.05). As shown in Table 5, these elderly patients showed a significantly lower mortality in almost all observed causes of death compared to the reference normal population during the total inclusion period.

Compared to the subgroups of younger patients (Figure 1), the >79 years age group comprised more patients in the later (*N*=170; 1997–2014) as compared to the early inclusion period (*N*=120; 1986–1996). Elderly patients treated in the early time period (1986–1996) had significantly decreased SMR in all causes of death compared to the reference population (SMR = 0.4, *P* < 0.05). There was a significant lower mortality due to diseases in the circulatory system (SMR = 0.3, *P* < 0.05) in older patients treated between 1986 and 1996.

## 4. Discussion

In the present study, the number of patients treated in the edentulous jaw is decreasing during the inclusion time which is in line with results from a study reported by Norderyd et al. where the frequency of middle-aged edentulous individuals in Sweden decreased over the last decades from 12% in 1983 to 0.3% in 2013 [20]. In the same time, the age distribution of edentulous patients demonstrated increasingly elderly patients (Figure 1), which was shown in this study and confirmed by several studies [1,20–23].

This large retrospective study shows a statistically significant association between edentulism and risk for all-cause mortality and for death due to the most common diseases. These results are in line with previous studies that has shown an association between missing teeth and mortality due to diseases in the circulatory system [8, 24, 25]. In this study, the time period for implant surgery was an important factor for the risk for all-cause mortality independent of age and gender. In all three age-related subgroups, patients treated in the earlier period (1986–1996) show lower risk for all-cause mortality compared to the patients treated in the later time period. The reason for this observation is unclear but could be that the edentulous patient nowadays may belong to a more vulnerable group of the population while the patients treated in the early period of implantology comprised more generally healthy patients who had lost teeth more due to lack of prophylactic care, socioeconomic, or attitude reasons [4]. However, tooth loss has been reported to be associated with increased risk for patient mortality [9, 26, 27] which was also confirmed in the present study, comparing the edentulous patient with the general population independent of time period.

Unlike the general Swedish population, the number of observed deaths due to diseases in the circulatory system was higher as compared to the number of deaths due to cancers in the group of patients treated in the later period of the study (1997–2014). The results show a statistically significant increase in mortality due to diseases in the circulatory system with later inclusion in younger patients (age range of 50–59 years). The present findings suggesting younger edentulous patients (aged <60 years) who have an increased risk for cardiovascular mortality are supported by a study from Brown [28] which conclude that complete edentulism prior to the age of 65 years also is associated with an increased risk of all-cause mortality.

These findings support the results of a previous study in which patients with periodontitis were shown to have an increased risk of cardiovascular and all-cause mortality [25]. In a recent published study from Nordendahl et al., [29] females younger than 65 years of age with severe periodontitis had an increased risk for myocardial infarction even after adjusting for different confounding factors such smoking, diabetes, education, and marital status. It is of interest to note that increased mortality due to diseases in the circulatory system has also been reported in patients with osteoarthritis who are undergoing knee replacement [15] and after total hip arthroplasty [30]. Both periodontitis and osteoarthritis are chronic inflammatory diseases [30–32], and periodontitis is the most common reason for tooth extraction in adults over 40 years of age [5, 33, 34]. Inflammation with high circulating concentrations of inflammation biomarkers (C-reactive protein or fibrinogen) is suggested to be associated with a subsequent risk for diseases in the circulatory system [35, 36]. The assumed increased inflammatory activity in younger edentulous patients may explain their higher rate of mortality due to diseases in the circulatory system, as compared to the reference population. Therefore, it could be suggested that edentulous middle-aged patients should be investigated for increased risk of future diseases in the circulatory system, in accordance to previous suggestions for younger patients provided with knee joint prostheses [15].

On the contrary, older edentulous patients (>79 years of age) treated with dental implants have shown increased longevity [14, 37] and the findings from this study with significant lower SMR in all investigated causes of death for these older patients could be associated with this observation.

A major limitation of the present study is the lack of adjustment for confounders, such as smoking and obesity, both of which increase the risk for diseases in the circulatory system. Also, the reason for edentulousness in the study patients remains unclear, and information about the socioeconomic status and education is missing. However, previous studies [9, 10, 38] suggested that the number of teeth was a predictor for mortality independent on socioeconomic status and lifestyle factors.

All the patients in the present study are restored with fixed implant-supported dental prostheses in the edentulous jaw. As known, there are other options for prosthetic treatment of the edentulous jaw-like removable dentures. Fukai et al. [39] suggested an association between mortality and complete dentures, where female subjects without dentures and less than 10 functional teeth show a significantly higher mortality rate. Another Japanese study [40] concluded that the use of dentures was associated with a decreased risk for mortality in persons aged 65 years or older. In a recent published systematic review from Gupta et al. [41], an association was found between prosthetic rehabilitation with a complete denture and mortality. This review concluded that denture wearing was associated with lower mortality compared to edentulous patients not using dentures. However, whether this is due to the use of the denture itself or due to the general capacity/motivation to wear a denture is not clear, and a causal relationship remains to be established.

## 5. Conclusions

Within the limitations of the present study, the following conclusions could be made for the mortality pattern of implant patients treated in the edentulous jaw between 1986 and 2014:

- For the total implant study group (*N*=3902 patients), the standardised mortality ratio (SMR) showed a generally increased mortality compared to the national reference population
- Patients treated early (between 1986 and 1997) showed a significantly lower mortality compared to the national reference population independent of age
- Younger patients (<60 years) treated with implants in the edentulous jaw late (between 1997 and 2014) showed an increased risk for mortality due to diseases in the circulatory system (CVD) compared to the national reference population of the same age
- Elderly patients treated with implants in the edentulous jaw (>79 years) showed significantly lower mortality for all causes of death independent of the time period of implant surgery compared to the national reference population

## Figures and Tables

**Table 1:** Mortality among 3902 (2142 females vs. 1760 males) treated edentulous patients undergoing treatment with dental implants 1986–2014 and deceased between 1986 and 2014.

Cause of death	Total number of deceased patients (*N*=2098)				Number of deceased males (*N*=1000)				Number of deceased females (*N*=1098)			
	Observed	Expected	SMR^*∗*^	95% CI^†^	Observed	Expected	SMR	95% CI	Observed	Expected	SMR	95% CI
Infectious diseases	32	19	1.6^‡^	1.1–2.2	17	8	2.2^‡^	1.1–3.2	15	12	1.2	0.6–1.9
Tumours	556	294	1.9^‡^	1.7–2.0	279	134	2.1^‡^	1.8–2.3	277	159	1.7^‡^	1.5–1.9
Endocrine/metabolic diseases	49	31	1.5^‡^	1.1–1.9	30	13	2.4^‡^	1.5–3.2	19	19	1.0	0.5–1.4
Mental illness	103	71	1.4^‡^	1.2–1.7	32	19	1.6^‡^	1.1–2.2	71	50	1.4^‡^	1.1–1.7
Diseases in the circulatory system	883	658	1.3^‡^	1.2–1.4	418	265	1.6^‡^	1.4–1.7	465	392	1.2^‡^	1.1–1.3
Diseases in the respiratory system	183	99	1.8^‡^	1.6–2.1	77	42	1.8^‡^	1.4–2.2	106	57	1.9^‡^	1.5–2.2
Diseases in the digestive system	50	43	1.2	0.8–1.5	27	18	1.5	0.9–2.1	23	25	0.9	0.5–1.3
All causes	2098	1377	1.5^‡^	1.5–1.6	1000	563	1.8^‡^	1.7–1.9	1098	813	1.35^‡^	1.3–1.4

^*∗*^SMR: standardised mortality ratio; ^†^95% CI, 95% confidence interval; ^‡^statistically significant.

**Table 2:** Mortality among 2496 (1384 females vs. 1112 males) treated edentulous patients undergoing treatment with dental implants 1986–1996 and deceased between 1986 and 1996.

Cause of death	Total number of deceased patients (*N*=245)				Number of deceased males (*N*=143)				Number of deceased females (*N*=102)			
	Observed	Expected	SMR^*∗*^	95% CI^†^	Observed	Expected	SMR	95% CI	Observed	Expected	SMR	95% CI
Infectious diseases	0	2	0.0		0	1	0.0		0	1	0.0	
Tumours	87	62	1.4^‡^	1.1–1.7	43	29	1.5	1.0–1.9	44	33	1.3	0.9–1.7
Endocrine/metabolic diseases	7	6	1.2	0.3–2.0	6	2	2.5	0.5–4.6	1	3	0.3^‡^	0.0–0.9
Mental illness	5	7	0.7	0.1–1.2	3	3	1.0	0.0–2.6	2	5	0.4^‡^	0.0–0.9
Diseases in the circulatory system	100	145	0.7^‡^	0.6–0.8	63	66	0.9	0.7–1.2	37	79	0.4^‡^	0.3–0.6
Diseases in the respiratory system	22	22	1.0	0.6–1.4	11	10	1.1	0.4–1.7	11	12	0.9	0.4–1.5
Diseases in the digestive system	7	9	0.8	0.2–1.4	5	4	1.2	0.2–2.4	2	5	0.4^‡^	0.0–0.9
All causes	245	278	0.9^‡^	0.8–0.9	143	127	1.1	0.9–1.3	102	150	0.7^‡^	0.5–0.8

^*∗*^SMR: standardised mortality ratio; ^†^95% CI, 95% confidence interval; ^‡^statistically significant.

**Table 3:** Mortality among 1406 (758 females vs. 648 males) treated edentulous patients undergoing treatment with dental implants 1997–2014 and deceased between 1997 and 2014.

Cause of death	Total number of deaths (*N*=459)				Number of male deaths (*N*=219)				Number of female deaths (*N*=240)			
	Observed	Expected	SMR^*∗*^	95% CI†	Observed	Expected	SMR	95% CI	Observed	Expected	SMR	95% CI
Infectious diseases	10	4	3.0^‡^	1.1–5.0	4	1	3.1	0.1–6.3	6	2	3.0	0.6–5.4
Tumours	129	37	3.5^‡^	2.9–4.1	68	17	4.0^‡^	3.0–4.9	61	19	3.2^‡^	2.4–4.1
Endocrine/metabolic diseases	10	2	4.3^‡^	1.6–6.9	6	2	3.4	0.6–6.1	4	3	1.4	0.1–2.9
Mental illness	21	6	3.7^‡^	2.1–5.2	6	3	1.9	0.4–3.4	16	9	1.7	0.8–2.5
Diseases in the circulatory system	185	44	4.2^‡^	3.6–4.9	88	33	2.7^‡^	2.1–3.2	97	55	1.8^‡^	1.4–2.1
Diseases in the respiratory system	36	7	5.4^‡^	3.7–7.2	14	6	2.5^‡^	1.2–3.8	22	8	2.9^‡^	1.7–4.1
Diseases in the digestive system	11	3	3.7^‡^	1.5–5.9	6	2	2.8	0.6–5.0	5	3	1.5	0.2–2.7
All causes	459	187	2.4^‡^	2.2–2.7	219	73	3.0^‡^	2.6–3.4	240	113	2.1^‡^	1.9–2.4

^*∗*^SMR: standardised mortality ratio; ^†^95% CI, 95% confidence interval; ^‡^statistically significant.

**Table 4:** Mortality among 389 treated edentulous patients undergoing treatment with dental implants who were 40–59 years of age at surgery and deceased younger than 60 years and treated and deceased during 1997–2014.

Causes of death	Total number of deceased patients (*N*=28)			
	Observed	Expected	SMR^*∗*^	95% CI^†^
Infectious diseases	0	—	—	—
Tumours	8	4	2.1	0.7–3.6
Endocrine/metabolic diseases	1	0.2	4.4	0–12.9
Mental illness	1	0.2	4.0	0.0–12.0
Diseases in the circulatory system	11	2	5.4^‡^	2.2–8.5
Diseases in the respiratory system	2	0.3	7.3	0–17.4
Diseases in the digestive system	1	0.4	2.3	0.0–6.9
All causes	28	10	2.7^‡^	1.8–3.9

^*∗*^SMR: standardised mortality ratio; ^†^95% CI, 95% confidence interval; ^‡^statistically significant.

**Table 5:** Mortality among 290 treated edentulous patients undergoing treatment with dental implants who were >79 years at surgery and treated and deceased during 1986–2014.

Causes of death	Total number of deceased patients (*N*=236)			
	Observed	Expected	SMR^*∗*^	95% CI^†^
Infectious diseases	3	6	0.5	0.0–1.1
Tumours	37	51	0.7^‡^	0.5–0.9
Endocrine/metabolic diseases	4	8	0.5^‡^	0.0–0.9
Mental illness	14	24	0.6^‡^	0.3–0.8
Diseases in the circulatory system	129	189	0.7^‡^	0.6–0.8
Diseases in the respiratory system	17	29	0.6^‡^	0.3–0.9
Diseases in the digestive system	3	10	0.3^‡^	0.0–0.6
All causes	236	362	0.6^‡^	0.5–0.7

^*∗*^SMR: standardised mortality ratio; ^†^95% CI, 95% confidence interval; ^‡^statistically significant.

## Data Availability

Due to ethical concerns, supporting data cannot be made openly available. The causes of death data used to support the findings of this study were supplied by the National Board of Health and Welfare in Sweden under license and therefore cannot be made openly available. Requests for general access to these data should be made to the National Board of Health and Welfare in Sweden (https://www.socialstyrelsen.se/english). Further information about the causes of death data and conditions for access are available at https://www.socialstyrelsen.se/statistics/statisticaldatabase/causeofdeath.
